# Effect of Diaphragmatic Resection Versus Stripping in Advanced Ovarian Cancer: Impact on Patient Complications in a Large Retrospective Cohort Study at a Tertiary Referral Center

**DOI:** 10.1245/s10434-025-18423-1

**Published:** 2025-10-04

**Authors:** Pietro Pasquini, Lucia Genovesi, Camelia Alexandra Coadă, Andi Stermasi, Francesco Mezzapesa, Stella Di Costanzo, Alessandro Bovicelli, Elisabetta Pia Bilancia, Enrico Fiuzzi, Giulia Mantovani, Pierandrea De Iaco, Anna Myriam Perrone

**Affiliations:** 1 Division of Oncologic Gynecology, IRCCS Azienda Ospedaliero-Universitaria di Bologna, Bologna, Italy; 2 Department of Medical and Surgical Sciences (DIMEC), University of Bologna, Bologna, Italy; 3 Department of Morpho-functional sciences, Iuliu Haţieganu University of Medicine and Pharmacy, Cluj-Napoca, Romania; 4 Fondazione Policlinico Universitario Campus Bio-Medico, Roma, Italy; 5 Department of Medical and Surgical Sciences, Division of Oncologic Gynecology, IRCCS Azienda Ospedaliero-Universitaria di Bologna, Bologna, Italy

**Keywords:** Ovarian cancer, Diaphragmatic surgery, Residual disease, Interval debulking surgery, Primary debulking surgery, Chemotherapy

## Abstract

**Background:**

Complete cytoreductive surgery is crucial in advanced ovarian cancer (OC) treatment. Diaphragmatic surgery, including stripping (DS) and resection (DR), is often necessary for optimal cytoreduction. However, postoperative complications and the timing of adjuvant chemotherapy initiation remain critical concerns. This study evaluates the impact of DR and DS on surgical outcomes, chemotherapy timing, and survival.

**Patients and methods:**

This retrospective, monocentric study analyzed 215 patients with International Federation of Gynecology and Obstetrics (FIGO) stage III–IV OC undergoing DS or DR between 2011 and 2023. Clinical, surgical, and survival data were collected; complications were graded using the Clavien–Dindo system. Statistical analysis included contingency and survival tests.

**Results:**

A total of 215 patients underwent diaphragmatic surgery: 122 patients (56.7%) underwent DR and 93 (43.3%) DS. No significant differences existed between groups regarding age, body mass index (BMI), histological subtype, American Society of Anesthesiologists (ASA) score, or primary/interval debulking surgery distribution (*p* = 0.122). DR was more common in patients with greater peritoneal disease (*p* = 0.003), higher pleural involvement (*p* = 0.002), and longer operative times (*p* = 0.018). Postoperatively, DR was associated with increased thoracic complications (87.7% versus 52.7%, *p* < 0.001), greater oxygen supplementation needs (55.7% versus 35.5%, *p* = 0.003), and elevated liver enzymes. However, no significant differences emerged in severe complications (*p* = 0.077), reoperation rates (*p* = 0.227), or time to chemotherapy initiation (*p* = 0.742). A decreasing trend in thoracostomy tube placement was observed since 2018. Progression-free and overall survival were similar between groups.

**Conclusions:**

Despite requiring greater intraoperative effort and resulting in higher postoperative morbidity, DR is not associated with an increased incidence of severe complications (grade 3+) or delayed chemotherapy initiation compared with DS. These findings support the feasibility of DR for achieving complete cytoreduction in advanced OC.

Ovarian cancer (OC) is the leading cause of death among women with gynecologic malignancies and the fifth most common cause of cancer-related mortality in women overall.^1^ Most cases (80%) are diagnosed at an advanced stage, leading to poor outcomes.^2^ Currently, the standard treatment consists of a combination of surgery and chemotherapy, administered in personalized sequences.^3,4^ Complete cytoreduction (CC0) consistently emerges as the most important prognostic factor and influential determinant of patient outcomes, irrespective of the timing of surgery [primary debulking surgery (PDS) versus interval debulking surgery (IDS)] or the number of cycles of chemotherapy.^5–7^ Consequently, the surgical management of ovarian cancer has significantly evolved over the past decade, shifting from procedures limited to the pelvic organs to more extensive cytoreductive surgeries involving the upper abdomen, such as diaphragmatic peritonectomy, splenectomy, and liver resection.^8–10^

The diaphragmatic region, particularly the right diaphragm, is a common site of tumoral spread, with approximately 40% of patients with advanced stage OC exhibiting visible disease on this peritoneal surface.^11^ In such cases, standard cytoreductive surgical procedures often include diaphragmatic peritoneal stripping (DS) or full-thickness diaphragmatic resection (DR), depending on the extent of disease and muscular layer infiltration.^12^ Recently, Tozzi et al. developed a three-tier classification system for diaphragmatic surgery and its associated morbidity, proposing it as a training benchmark.^13^ In current surgical practice, adjunctive technologies such as PlasmaJet^®^ and Argon Beam Coagulation^®^ are commonly used to facilitate safe and complete tumor resection, particularly in delicate anatomical sites and in patients treated with neoadjuvant chemotherapy.^14,15^

Thoracic complications are reported in 43–75% of cases in the literature, including pleural effusion (37%), pulmonary embolism (5%), pneumothorax (4%), and pulmonary infection (2%).^16^ Among these, pneumothorax and pleural effusion are the most commonly investigated complications, as they may be preventable through intraoperative thoracic drainage,^17^ although this approach remains debated.^12^ These complications may lead to delays in the initiation of adjuvant chemotherapy.^18^ The specific impact of DS and DR on postoperative recovery and timing of chemotherapy remains unexplored but deserves further investigation, given that delayed chemotherapy beyond certain thresholds is associated with significantly worse oncologic outcomes in ovarian cancer.^19,20^

The primary aim of this study was to evaluate the impact of diaphragmatic resection (DR) versus stripping (DS) on postoperative complications and time to chemotherapy initiation. Secondary objectives included the analysis of survival outcomes and the assessment of intraoperative thoracic drainage in DR, considering its debated role in preventing thoracic complications.

## Patients and Methods

### Study Design

This was a retrospective, cohort study conducted at the Division of Oncologic Gynecology IRCCS Azienda Ospedaliero-Universitaria di Bologna, Italy, and approved by the Ethics Committee of the Area Vasta Emilia Centro (AVEC) with code number 51/2024/Oss/AOUBo. Inclusion criteria were: (1) diagnosis of advanced stage OC [International Federation of Gynecology and Obstetrics (FIGO) stage III–IV]; (2) histologically confirmed OC of any histological type and grade; and (3) only patients who underwent debulking surgery with DS and/or DR between 2011 and 2023. Exclusion criteria were: (1) diaphragmatic fulguration of nodules without major surgery (e.g., argon beam coagulation, electrosurgery, or other ablative techniques) and (2) patients with incomplete clinical and/or surgical data.

The participants were identified through the institutional surgical database and consecutively reviewed on the basis of inclusion criteria. To mitigate selection bias, the largest possible number of consecutive eligible patients within the study period was included. However, due to the retrospective nature of the study, reporting and classification bias cannot be entirely excluded. The Strengthening the Reporting of Observational Studies in Epidemiology (STROBE) guidelines were followed.

### Patient Selection and Data Collection

Clinical, pathological, surgical, and follow-up data were retrieved from the electronic medical records of all enrolled patients. Data were extracted from the institutional surgical database by two independent reviewers, with final review and re-opening of records for complication verification when needed. Collected variables included age at diagnosis, body mass index (BMI), serum tumor markers, radiological imaging [typically thoracoabdominal computed tomography (CT) scan], gastroscopy, colonoscopy, histological subtype, FIGO stage, diagnostic laparoscopy to assess histology and operability, ascites, pleural effusion, neoadjuvant chemotherapy, and laboratory tests. Surgical data included surgical radicality, distribution of diaphragm implants, type of diaphragm surgery, occurrence of pleural cavity perforation, use of mesh during diaphragm repair, placement of a prophylactic chest tube, operative time, residual disease, and surgery-to-chemotherapy interval. Additional details included intraoperative transfusions, postoperative intensive care unit (ICU) admission, oxygen therapy, pleural effusion symptoms, and evacuation procedures with dates. Data on drainage duration and fluid volume during thoracic evacuation procedures were also recorded.

### Surgical Diaphragmatic Technique

Diaphragmatic surgery was performed by the Cliby technique.^21^ Briefly, a retractor device (Sattler Retractor^®^, Medizintechnik Sattler GmbH, Königsee, Germany) was positioned to elevate the costal margin and tension the tissues. The falciform, coronary, and triangular ligaments of the liver were resected to achieve liver mobilization. Depending on the muscle infiltration during peritonectomy, either DS or DR was performed with a monopolar scalpel. In case of DR, following trans-diaphragmatic chest exploration to rule out pleural spread, the defect was closed using a monofilament non-resorbable stitches applied as two running sutures tied in the middle. To prevent iatrogenic pneumothorax, a suction tube was carefully inserted and removed while the final sutures were secured. After closure, a bubble test was performed to detect any defect. The decision to use a prophylactic chest tube was made by the operating surgeon on the basis of the extent of diaphragmatic involvement and patient comorbidities. If a tension-free suture was unachievable due to defect size or tissue retraction, a GORE-TEX mesh (Gore^®^ Dualmesh^®^, W. L. Gore & Associates, Newark, Delaware, USA) was placed. In cases of bilateral diaphragmatic surgery or accidental diaphragm opening, the patient was allocated to the more invasive group (DR).

### Perioperative Complications

Perioperative morbidity and mortality were recorded and graded according to the Clavien–Dindo classification. Chest radiographs were taken at 24 h and 48 h after surgery and additional imaging (X-ray or CT scan) was performed depending on the clinical condition of the patient. In patients with preoperative pleural effusion, fluid volume was assessed by comparing preoperative and postoperative chest X-rays. Pleural effusion was defined as severe if it occupied more than half of the chest radiographs; all other cases were classified as modest or mild.

### Statistical Analysis

Statistical analysis was performed in R Statistical Software (version 4.3.2; R Core Team 2021).^22^ Descriptive statistics were provided for all variables, in the form of absolute numbers and frequency for nominal variables and mean and standard deviation (SD) or median and interquartile range (IQR) for continuous variables. Depending on the type of variable, chi-squared test, Fisher’s exact test, Student’s *t*-test, and Mann-Whitney *U* test were used to analyze the differences between patient groups. Logistic regression was applied to model dichotomous outcome variables. Progression-free survival (PFS) and overall survival (OS) were estimated using the Kaplan–Meier method and significance among groups was tested using the log-rank test. Cox proportional hazards was used to estimate the impact of variables of interest on OS in both univariable and multivariable models. Results with a *p* value ≤ 0.05 were considered statistically significant. A post hoc power analysis was conducted to assess sample size adequacy for the main outcomes: postoperative complications (overall and severe, Clavien–Dindo ≥ 3) and time to chemotherapy. Effect sizes, alpha level (0.05), and power estimates are detailed in the Supplementary Material.

## Results

### Surgical Procedures

**Fig. 1:**
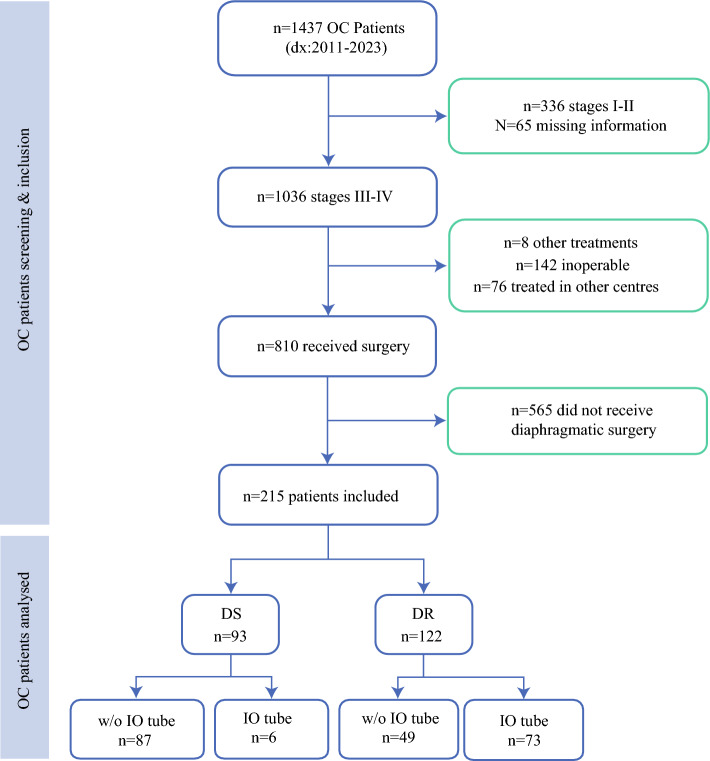
Flowchart showing the selection of the patients for this study. DS: Diaphragmatic Stripping; DR: Diaphragmatic Resection; IO: Intra-Operative; w/o: without

Across a period of 12 years, 810 patients with advanced OC underwent surgery, among them 215 (26.5%) met the inclusion criteria. Among these, 144 (67.0%) received PDS, while 71 patients (33.0%) underwent IDS (Fig. 1).

**Fig. 2:**
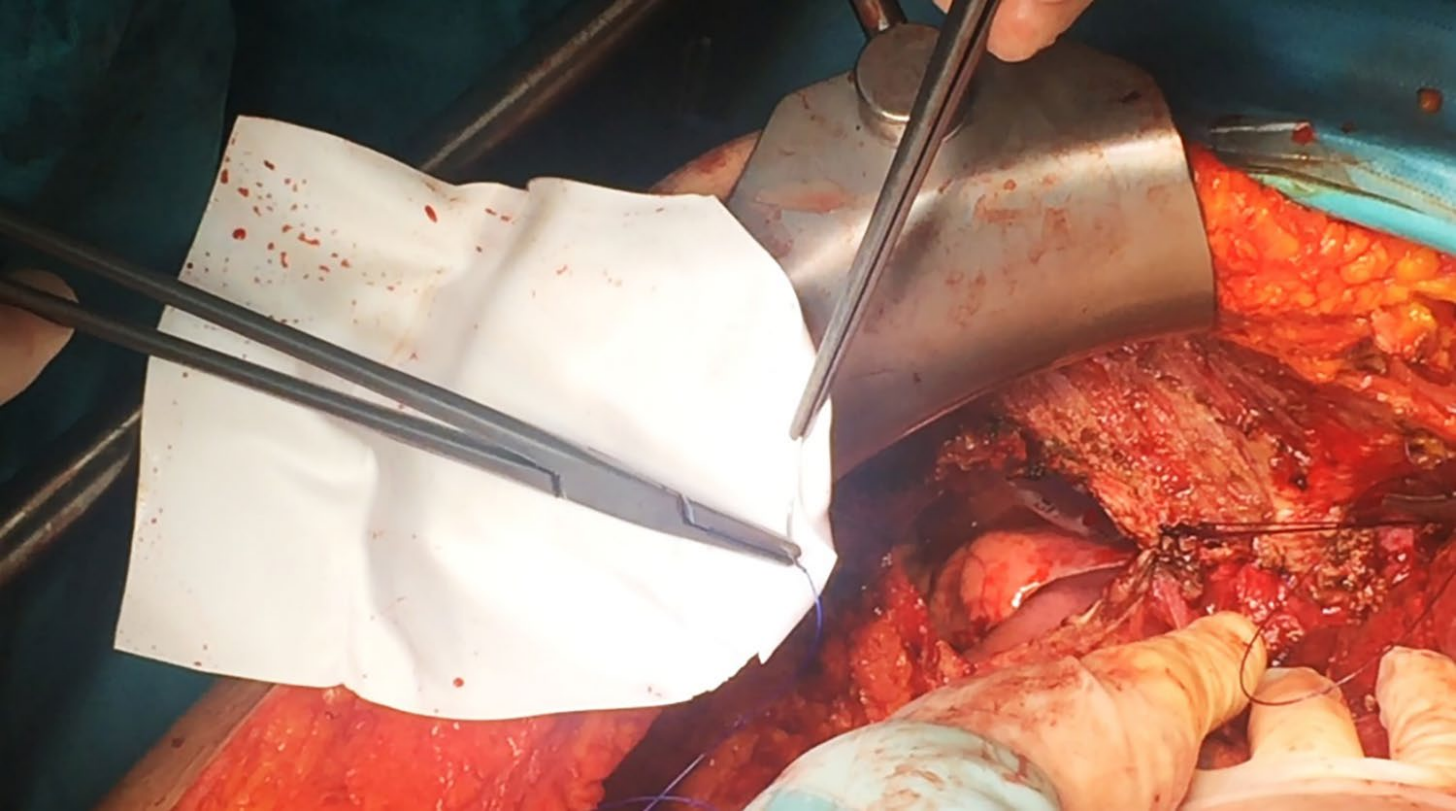
Diaphragmoplasty with a Gore-Tex prosthesis in a DR patient requiring diaphragmatic repair

A total of 122 DR procedures (56.7%) and 93 DS procedures (43.3%) were performed. Accidental diaphragmatic openings during stripping were included in the DR group, comprising 29 out of 122 cases (23.8%). One case in the DR group (0.8%) required diaphragmoplasty using a Gore-Tex prosthesis (Fig. 2). All surgeries were performed via a midline xipho-pubic laparotomy.

### Intraoperative Characteristics

The DR procedure was more frequently performed in patients with stage IV disease (38 versus 12 cases *p* = 0.002) and those with higher PCI (*p* = 0.003). Consequently, DR surgeries were associated with longer durations (*p* = 0.018) and more complex procedures (*p* = 0.001), as well as increased need for intraoperative blood transfusions (*p* = 0.002) (Table 1). A sub-analysis of surgical procedures showed that pelvic and para-aortic lymphadenectomies, rectosigmoid resections, and ileostomies were significantly more frequent in the DR group, while other procedures were evenly distributed between the two groups (Supplementary Table 5).

**Table 1:** Surgical characteristics of the patients included in the study

Variable		Total *N* = 215 (100%)	All patients		*p* Value
			DS *N* = 93 (43.3%)	DR *N* = 122 (56.7%)	
Type of surgery, *N* (%)	PDS	144 (67)	57 (61.3)	87 (71.3)	0.122
	IDS	71 (33)	36 (38.7)	35 (28.7)	
Ascites before surgery, *N* (%)		154 (71.6)	65 (69.1)	89 (73.5)	0.622
	> 3.5 L	14 (6.5)	6 (6.5)	8 (6.6)	0.980
Pleural effusion before surgery, *N* (%)		26 (12.09)	13 (13.97)	13 (10.65)	0.459
PCI at staging laparoscopy, *median* (*IQR*)		16 (11, 20)	14 (10, 18.5)	17 (12, 20)	0.070
PCI at debulking surgery, *median* (*IQR*)		12 (8, 18)	11 (7, 14.75)	15 (9, 20)	0.003
FIGO stage at diagnosis, *N* (%)	IIIB/IIIC	165 (76.74)	80 (86.02)	82 (67.21)	0.002
	IV	50 (23.26)	12 (12.90)	38 (31.14)	
Completeness of cytoreduction, *N* (%)	CC0	175 (81.4)	76 (80.85)	99 (81.82)	0.942
	CC1	27 (12.56)	13 (13.83)	14 (11.57)	
	CC2	8 (3.72)	3 (3.19)	5 (4.13)	
	CC3	5 (2.33)	2 (2.13)	3 (2.48)	
Aletti surgical complexity score,* median* (*IQR*)		10 (7, 12)	9 (7, 11)	10 (9, 12)	0.001
Laterality of diaphragmatic surgery, *N* (%)	Right	174 (80.9)	74 (79.6)	100 (82)	0.067
	Left	4 (1.9)	4 (4.3)	0 (0)	
	Bilateral	37 (17.2)	15 (16.1)	22 (23.7)	
Intraoperative transfusions, *median* (*IQR*)		0 (0, 2)	0 (0, 2)	1 (0, 2)	0.002
Number of transfusion bags, *median* (*IQR*)		2 (1, 4)	2 (0, 2)	2 (2, 4)	< 0.001
Operative time, min, *median* (*IQR*)		245 (195.75, 289.25)	225 (189.75, 270)	255 (216.25, 300)	0.018

*DS* diaphragmatic stripping, *DR* diaphragmatic resection, *n* number of cases; *PDS* primary debulking surgery, *IDS* interval debulking surgery, *PCI* Peritoneal Cancer Index

### Postoperative Outcomes

A total of 170 postoperative complications were reported in 215 surgeries, corresponding to an overall rate of 79.1%. The DR group had a higher complication rate, affecting 91% of cases (111 out of 122 cases) compared with 63.4% in the DS group (59 out of 93 cases, *p* < 0.001). Thoracic complications showed a similar pattern, occurring in 72.6% of all patients (156 cases). They were more frequent in the DR group than in the DS group (87.7% versus 52.7%, *p* < 0.001). The most common thoracic complications were moderate pleural effusion and transient postoperative pneumothorax (Table 2).

**Table 2:** Postoperative complications and clinical features

Variable	All patients			
	Total *N* = 215 (100%)	DS *N* = 93 (43.3%)	DR *N* = 122 (56.7%)	*p*-Value
Total complications (DINDO), *N* (%)	170 (79.1)	59 (63.4)	111 (91)	< 0.001
Grade 3+,* N* (%)	52 (24.2)	17 (18.3)	35 (28.7)	0.077
Thoracic complication, *N* (%)	156 (72.6)	49 (52.7)	107 (87.7)	< 0.001
Pleural effusion (G 3a),* N* (%)	27 (12.6)	8 (8.6)	19 (15.6)	0.652
Pneumothorax (G 3a),* N* (%)	1 (0.5)	1 (1.1)	0 (0)	0.044
Oxygen administration,* N* (%)	101 (47.0)	33 (35.5)	68 (55.7)	0.003
Pulmonary embolism,* N* (%)	4 (1.9)	2 (2.2)	2 (1.6)	0.784
Postoperative thoracentesis/drainage,* N* (%)	28 (13)	9 (9.7)	19 (15.6)	0.130
AST after surgery,* median* (*IQR*)	102 (73, 166.25)	89 (68, 126)	115 (78, 198)	0.033
ALT after surgery,* median* (*IQR*)	85 (55, 133)	71.5 (53.25, 110.5)	96 (59, 147)	0.048
Relaparotomy (< 30 days),* N* (%)	23 (10.7)	8 (8.6)	17 (13.9)	0.227
Time to start chemotherapy, days,* median* (*IQR*)	47 (40, 58)	48.5 (41, 59.3)	47 (38, 55)	0.742

*DS* diaphragmatic stripping, *DR* diaphragmatic resection, *n* number of cases, *G* grade

Overall, severe complications (Clavien–Dindo grade ≥ 3) were more frequent in the DR group compared with the DS group, although the difference did not reach statistical significance (28.7% versus 18.3%, *p* = 0.077). However, the incidence of severe postoperative thoracic complications was similar between the groups: pleural effusions occurred in 15.6% of DR cases and 8.6% of DS cases (*p* = 0.652), and only one case of pneumothorax was recorded in the DS group (Table 2). Postoperative fluid evacuation was required in 28 cases (13%), either through chest drainage, via ultrasound-guided pleural catheter placement or a single J drain (Pleur-evac^®^, Teleflex Medical), or thoracentesis. No significant differences were observed between groups (*p* = 0.130) (Table 2). Oxygen supplementation was required in 47% of patients (101 cases), with a significantly higher incidence in the DR group compared with the DS (55.7% versus 35.5% respectively; *p* = 0.003). There was no significant difference in pulmonary embolism occurrence (*p* = 0.784) (Table 2).

Postoperative liver enzyme levels were higher in patients who underwent DR, with significantly increased AST/GOT (*p* = 0.033) and ALT/GPT (*p* = 0.048) levels (Table 2). A subgroup analysis excluding patients who underwent hepatic resections showed that only AST/GOT remained significantly elevated, while ALT/GPT levels did not show a significant increase (Table 2).

A total of 23 reoperations (10.7%) were performed within 30 days of surgery, with no significant difference between the DR group (13.9% versus 8.6% respectively, *p* = 0.227). No deaths occurred within 30 days (Table 2).

**Fig. 3:**
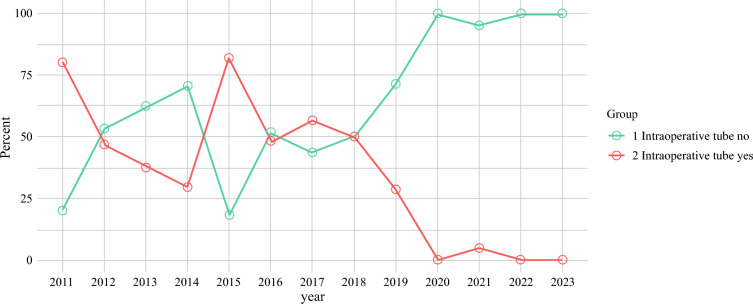
Trend in intraoperative thoracostomy tube placement from 2011 to 2023, showing a marked decrease in usage after 2018

The use of intraoperative thoracostomy tubes declined significantly after 2018, without affecting thoracic complication rates (Fig. 3).

A subanalysis of DR patients treated with and without intraoperative thoracostomy tube found no differences in surgical complications, postoperative pleural effusion or pneumothorax (Table 3). Patients with intraoperative thoracostomy tube presented a higher rate of oxygen supplement (65.8% versus 40.8%, respectively, with and without IO tube, *p* = 0.007) and a higher rate of reoperation within 30 days (19.2% versus 6.1%, respectively with or without IO tube; *p* = 0.041). Additionally, despite intraoperative drainage, ten DR patients required drainage and/or thoracentesis, specifically: in three cases (30%), to complications occurred into the contralateral; in three cases (30%), due to recurrence of ipsilateral pleural effusion after 24–72 h after the removal of the drainage; in two cases (20%), a pig-tail catheter was placed due to malfunctioning thoracic drainage; and in two cases (20%), thoracic drainage was repositioned during relaparotomy for hemoperitoneum near the diaphragm that had undergone DR.

**Table 3:** Postoperative complications and clinical features for DR patients only

Variable	DR-only patients		*p*-Value
	w/o IO tube *N* = 49 (40.2%)	with IO tube *N* = 73 (59.8%)	
Patients complicated,* N* (%)	44 (89.8)	67 (91.8)	0.707
Grade 3+,* N* (%)	12 (24.5)	23 (31.5)	0.400
Thoracic complication, *N* (%)	42 (85.7)	65 (89)	0.441
Pleural effusion (G 3a),* N* (%)	9 (18.4)	10 (13.7)	0.479
Pneumothorax (G 3a),* N* (%)	0 (0)	0 (0)	1
Pulmonary embolism,* N* (%)	0 (0)	2 (2.7)	0.515
Oxygen administration,* N* (%)	20 (40.82)	48 (65.8)	0.007
Postoperative thoracentesis/drainage,* N* (%)	9(18.4)	10(13.7)	0.490
Reoperation (< 30 days), *N* (%)	3 (6.1)	14 (19.2)	0.041
Time to start chemotherapy, days, *median* (*IQR*)	47 (41, 56)	45.5 (37.7, 54)	0.513

*DS* diaphragmatic stripping, *DR* diaphragmatic resection, *n* number of cases, *G* grade

### Adjuvant Chemotherapy

The median time of surgery to the start of adjuvant chemotherapy was similar for both DR and DS groups (48.5 versus 47 days respectively, *p* = 0.742) (Table 2). Within the DR group, the presence of an intraoperative thoracostomy tube did not affect the timing of chemotherapy initiation (*p* = 0.513) (Table 3).

### Oncological Outcomes

**Fig. 4:**
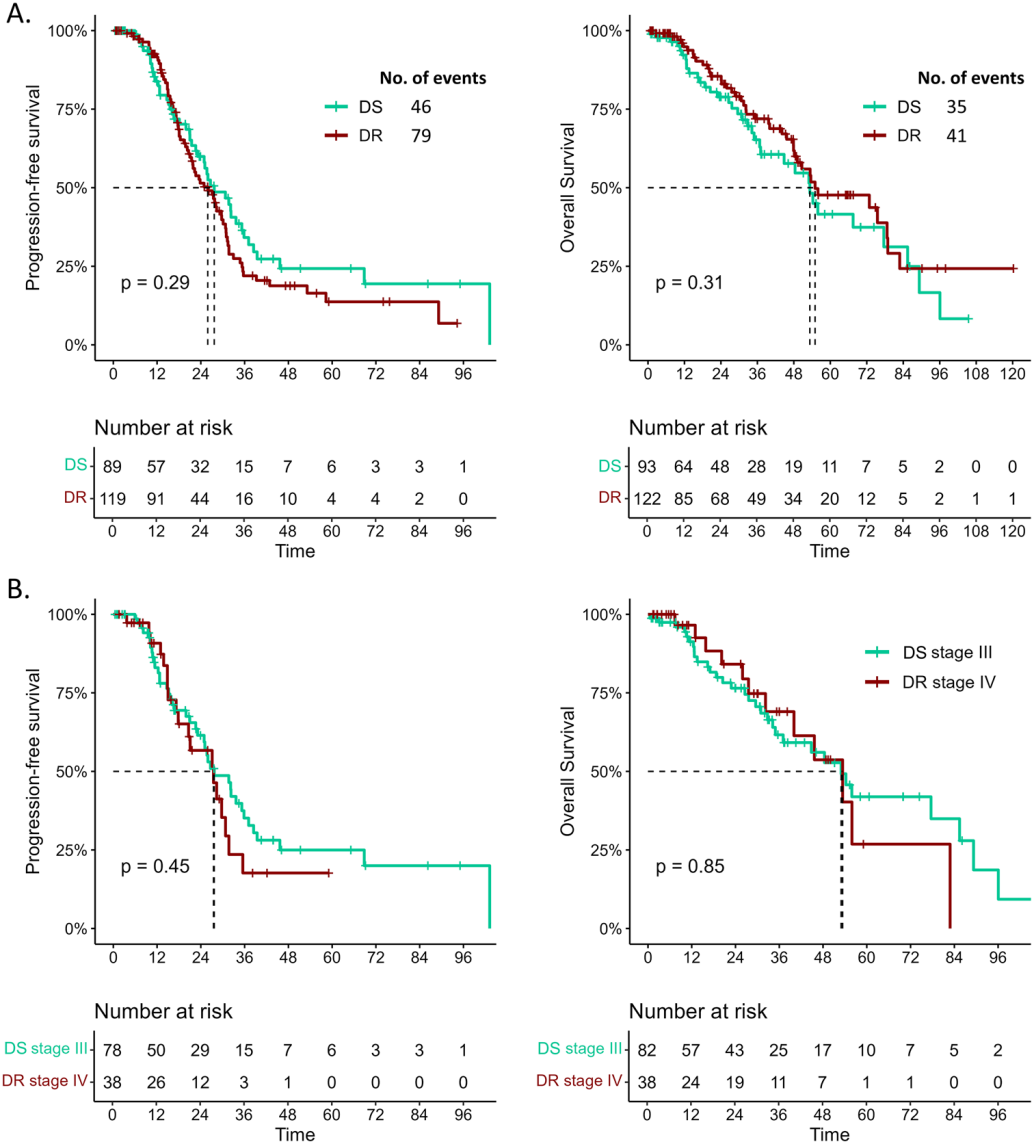
Kaplan-Meier curves showing the patients PFS (**A**) and OS (**B**) grouped by the type of diaphragmatic surgery. DS: diaphragmatic peritoneal stripping; DR: diaphragmatic resection.

With a median follow-up of 37.5 months (IQR 11.2, 66.6), there was no significant difference in patient prognosis between the two types of procedure in terms of PFS and OS (Fig. 4). Notably, survival outcomes were not influenced by the thickness of cancer invasion, remaining similar between patients with pleural invasion (stage IV) and those without (stage III). Cox proportional hazards analysis revealed that the type of diaphragmatic surgery had no impact on either PFS (*p* = 0.293) or OS (*p* = 0.314) (Supplementary Table 6).

## Discussion

To the best of our knowledge, this is the largest study on diaphragmatic surgery in ovarian cancer and the first to evaluate the impact of a more invasive procedure, such as DR, on post-surgical recovery time. Although more aggressive compared with DS, it does not prolong recovery and ultimately does not delay chemotherapy start.

### Comparison Between DR and DS

This study compares postoperative outcomes and complications between two types of procedures: DR and DS in patients undergoing OC surgery. Our results reveal significant differences in complications between the two groups, particularly thoracic issues. The DR group demonstrated a higher incidence of complications, including a greater frequency of pleural effusion and the need for oxygen supplementation. Additionally, DR patients exhibited a higher rate of liver enzyme abnormalities and required more intraoperative interventions, highlighting the complexity of the procedure and the associated risks. The higher complication rates observed in the DR group are likely attributable to the nature of the procedure itself. DR was more commonly performed in patients with advanced-stage disease (stage IV), who are generally at higher risk for intraoperative challenges and postoperative complications.^24^ These patients typically present with more extensive disease and higher PCI scores, which may contribute to prolonged and more complex surgeries. This is consistent with prior studies that have shown that advanced disease stages and high PCI scores are associated with increased surgical difficulty and poorer postoperative outcomes.^25^ The increased incidence of intraoperative blood transfusions in the DR group further supports this notion, indicating greater intraoperative morbidity. Thoracic complications, including pleural effusion and pneumothorax, were notably more prevalent in the DR group. The higher incidence of these complications in DR patients (87.7% versus 52.7% in DS patients,* p* < 0.001) may be related to the more invasive nature of the diaphragmatic repair. As a direct consequence of DR, unlike DS, the thoracic serosa (pleura) is opened, impairing pleural fluid clearance and leading to effusion. Additional damage to venous and lymphatic pathways contributes to vascular and lymphatic stasis, while the local inflammatory response from reparative processes further promotes pleural effusion, consistently seen, even if minimal, on postoperative imaging. Moreover, the DR group required oxygen supplementation more frequently, with more than half of DR patients (55.7%) needing supplemental oxygen postoperatively, compared with only 35.5% of DS patients (*p* = 0.003). This suggests that the DR procedure may be more likely to cause respiratory complications or exacerbate preexisting conditions, possibly due to the extent of diaphragmatic manipulation during the surgery. However, in line with previous findings,^12,17^ this difference did not translate into a significant increase in the incidence of severe thoracic complications (Clavien–Dindo grade 3+) or pulmonary embolism between the two groups, which remained low overall.

Liver function abnormalities were more common in the DR group, with elevated transaminase levels. These findings could be indicative of liver stress or minor hepatic injury, which may be a consequence of extensive surgical manipulation, especially in patients with advanced disease. Although liver enzymes were elevated when compared between the two groups, these values did not result in clinically significant liver failure or necessitate further interventions, suggesting that while liver stress may be a transient issue, it is not a major complication. In terms of reoperations, the rate was not significantly different between the DR and DS groups, with similar reoperation rates in both. This is a crucial finding, suggesting that despite the higher complication rates in the DR group, these patients did not require reoperation more frequently than those who underwent DS, likely due to the meticulous management of postoperative complications.

In our clinical practice, we frequently use Argon Plasma Coagulation^®^ (Erbe, USA) for small, superficial diaphragmatic implants, as shown effective in the literature.^14,15,23^ However, due to its superficial action and limited ability to assess full-thickness invasion, it is reserved for selected cases with small lesions and high likelihood of achieving CC0. Patients treated only with ablative techniques (e.g., fulguration or biopsy) were excluded to avoid selection bias.

### Role of Intraoperative Thoracostomy Tube in DR

The use of intraoperative thoracostomy tubes decreased after 2018, but thoracic complications remained unchanged. This trend may reflect improvements in surgical technique, surgical experience, or postoperative care over time. Additionally, our analysis of patients with and without intraoperative thoracostomy tubes showed no significant differences in complications, suggesting that routine use does not prevent pleural effusion or pneumothorax. However, ten DR patients still required interventional procedures such as drainage or thoracentesis, emphasizing the need for careful postoperative monitoring, particularly in complex surgeries such as DR.

As the indication for intraoperative thoracic drainage remains debated, in our clinical practice it is reserved for selected high-risk cases (e.g., preexisting pulmonary conditions, stage IV disease with pleural effusion, or extensive resections), introducing a potential selection bias. Additionally, the presence of a thoracic drain may impair pulmonary excursion and limit physiological respiration, possibly contributing to increased oxygen requirements. Therefore, the higher rate of oxygen administration in the DR group may reflect selection bias rather than a direct effect of drainage. Finally, reoperations were mostly attributed to major postoperative bleeding or anastomotic dehiscence, and therefore likely related to factors other than the diaphragmatic surgical technique.

### Oncological Outcomes and Chemotherapy Timing

A strength of this study is the comparable timing of adjuvant chemotherapy between the DR and DS groups, suggesting that the type of surgery did not significantly impact the initiation of subsequent treatments. Timely chemotherapy initiation is crucial for improving outcomes, making this finding oncologically significant. The presence of an intraoperative thoracostomy tube did not influence the timing of chemotherapy initiation, further supporting the notion that thoracic interventions during surgery do not delay postoperative care.^2^

Finally, in terms of oncological outcomes, no significant difference in PFS  OS was found between DR and DS patients. This supports other studies suggesting that the correct surgical approach with complete removal of the disease improves oncological outcomes.^17^ Similar survival rates after 37.5 months highlight the role of surgical expertise and postoperative care in managing advanced-stage OC, regardless of the diaphragmatic surgery performed.

### Strengths and Limitations

The study has several strengths: it encompasses the largest cohort described in the literature regarding diaphragmatic surgery for ovarian cancer (215 patients) who were treated according to international guidelines. Additionally, the study was conducted in a high-volume center specializing in ovarian cancer, with all procedures performed by an expert surgical team. These factors enhance the reliability of the results. Moreover, for the first time, we demonstrate no difference in the surgery-to-chemotherapy interval between the two techniques. Despite these strengths, we acknowledge some limitations, including the retrospective nature of the study and prolonged enrollment period and the heterogeneity of the DR group, which also included patients undergoing diaphragmatic stripping with incidental full-thickness resections. However, we made comparisons between two techniques for which treatment progress was observed in both groups over time. Although the procedures were performed by experienced surgeons in a high-volume center, the findings may not be directly applicable to centers with different levels of expertise or resources.

## Conclusions

While DR has higher complication rates, it does not significantly impact the long-term oncological outcomes compared with DS. These findings suggest that although DR is technically more demanding and typically performed in the context of more extensive cytoreductive procedures, it does not appear to be associated with increased intraoperative mortality or delayed initiation of chemotherapy in this cohort, although the number of patients does not allow for definitive conclusions. Future studies focusing on optimizing surgical techniques and postoperative management may help reduce the complication rates associated with DR, improving patient outcomes without sacrificing survival.
