# Correction: Effect of Diaphragmatic Resection Versus Stripping in Advanced Ovarian Cancer: Impact on Patient Complications in a Large Retrospective Cohort Study at a Tertiary Referral Center

**DOI:** 10.1245/s10434-025-18589-8

**Published:** 2025-10-09

**Authors:** Pietro Pasquini, Lucia Genovesi, Camelia Alexandra Coadă, Andi Stermasi, Francesco Mezzapesa, Stella Di Costanzo, Alessandro Bovicelli, Elisabetta Pia Bilancia, Enrico Fiuzzi, Giulia Mantovani, Pierandrea De Iaco, Anna Myriam Perrone

**Affiliations:** 1 Division of Oncologic Gynecology, IRCCS Azienda Ospedaliero-Universitaria di Bologna, Bologna, Italy; 2 Department of Medical and Surgical Sciences (DIMEC), University of Bologna, Bologna, Italy; 3 Department of Morpho-functional sciences, Iuliu Haţieganu University of Medicine and Pharmacy, Cluj-Napoca, Romania; 4 Fondazione Policlinico Universitario Campus Bio-Medico, Roma, Italy; 5 Department of Medical and Surgical Sciences, Division of Oncologic Gynecology, IRCCS Azienda Ospedaliero-Universitaria di Bologna, Bologna, Italy

**Correction: Ann Surg Oncol** 10.1245/s10434-025-18423-1

In the original online version of this article, Andi Stermasi's family name was incorrectly spelled. The original article was corrected.

